# Both mechanism and age of duplications contribute to biased gene retention patterns in plants

**DOI:** 10.1186/s12864-016-3423-6

**Published:** 2017-01-06

**Authors:** Hugo V. S. Rody, Gregory J. Baute, Loren H. Rieseberg, Luiz O. Oliveira

**Affiliations:** 1 Department of Biochemistry and Molecular Biology, Universidade Federal de Viçosa, Viçosa, 36570-900 Minas Gerais Brazil; 2 Department of Botany, University of British Columbia, Vancouver, BC V6T 1Z4 Canada

**Keywords:** Biased gene retention, Polyploidy, Transcription factors, Whole-genome duplication

## Abstract

**Background:**

All extant seed plants are successful paleopolyploids, whose genomes carry duplicate genes that have survived repeated episodes of diploidization. However, the survival of gene duplicates is biased with respect to gene function and mechanism of duplication. Transcription factors, in particular, are reported to be preferentially retained following whole-genome duplications (WGDs), but disproportionately lost when duplicated by tandem events. An explanation for this pattern is provided by the Gene Balance Hypothesis (GBH), which posits that duplicates of highly connected genes are retained following WGDs to maintain optimal stoichiometry among gene products; but such connected gene duplicates are disfavored following tandem duplications.

**Results:**

We used genomic data from 25 taxonomically diverse plant species to investigate the roles of duplication mechanism, gene function, and age of duplication in the retention of duplicate genes. Enrichment analyses were conducted to identify Gene Ontology (GO) functional categories that were overrepresented in either WGD or tandem duplications, or across ranges of divergence times. Tandem paralogs were much younger, on average, than WGD paralogs and the most frequently overrepresented GO categories were not shared between tandem and WGD paralogs. Transcription factors were overrepresented among ancient paralogs regardless of mechanism of origin or presence of a WGD. Also, in many cases, there was no bias toward transcription factor retention following recent WGDs.

**Conclusions:**

Both the fixation and the retention of duplicated genes in plant genomes are context-dependent events. The strong bias toward ancient transcription factor duplicates can be reconciled with the GBH if selection for optimal stoichiometry among gene products is strongest following the earliest polyploidization events and becomes increasingly relaxed as gene families expand.

**Electronic supplementary material:**

The online version of this article (doi:10.1186/s12864-016-3423-6) contains supplementary material, which is available to authorized users.

## Background

Gene duplication has long been viewed as a key driver of biological complexity in Eukaryotes [1–4]. Duplicate genes mainly arise via small-scale tandem or segmental duplication events or via large-scale whole genome duplications (WGDs). The latter are especially common in plants [5, 6]. Indeed, comparative genomic studies indicate that all extant seed and flowering plants have experienced one or more WGDs in their evolutionary history [7–12].

Following gene duplication (whether via tandem, segmental or WGD events), most duplicate copies become pseudogenes (i.e. lose their function) or are lost entirely due to deletions [13]. This is expected because of relaxed purifying selection due to functional redundancy. Large-scale deletions are especially common following WGDs, as the neopolyploid returns back to its ancestral diploid condition, a process referred to as diploidization. Nevertheless, some gene duplicates are retained, and these surviving duplicates appear to contribute importantly to the evolution of biological complexity and phenotypic novelty, in part because such genes are less constrained evolutionarily than are single copy genes [14–16].

Several models have been put forward to explain how duplicate genes avoid pseudogenization, as well as to account for why some duplicate genes are retained and others are not [17]. These include (1) neofunctionalization, in which one of the duplicates (i.e. paralogs) acquires a new function; (2) subfunctionalization, in which ancestral function is partitioned among paralogs [1]; (3) relative dosage, in which duplicate genes are retained (or lost) to avoid dosage imbalances [18, 19]; and (4) absolute dosage, in which the fixation of duplicate genes is due to selection favoring an increase in gene dosage [20] or metabolic flux [21].

In this paper, we focus on the predictions of the relative dosage model, also known as the Gene Balance Hypothesis (GBH) [18, 22], as this hypothesis has garnered the most support from real data [19, 23–26]. According to the GBH, genes with a large number of interactions (i.e., “connected genes”) should be retained disproportionately following WGD events thereby maintaining optimal stoichiometry among their products; when a WGD event occurs, all genes are duplicated simultaneously and so relative gene dosage should not change. In small-scale duplications (e.g., tandem events), the increased dosage of a single, highly connected gene could result in decreased fitness, or even in lethality. Therefore, connected genes are expected to be differentially lost following small-scale duplications. Conversely, genes that work alone or have few interactions, such as those involved in disease resistance, are more likely to be retained following tandem duplications.

Patterns of gene retention in *Arabidopsis thaliana* are largely consistent with GBH predictions. For example, highly connected genes such as transcription factors have been preferentially retained after WGDs in *A. thaliana*, but disproportionately lost following small-scale duplications [23, 24]. The bias towards regulatory genes chiefly derives from duplicates of intermediate age (circa 50–70 mya), which are mainly WGD-associated [13]. Similar findings have been reported for poplar [26] and rice [25]. In contrast, paleologs (paralogs arising from WGD events) in the Compositae family are enriched for genes annotated to structural components or cellular organization gene ontology (GO) categories, while genes involved with transcription appear to be significantly under-represented [10]. In *A. thaliana* and *Sorghum bicolor*, both WGD and tandem mechanisms of duplication are associated with paralogs involved in high metabolic flux networks [21], an observation best predicted by the absolute dosage model.

In addition to mechanism of duplication, the fate of paralogs may be influenced by genetic background, various environmental factors, epigenetic effects, genetic drift, and the mechanism of gene dosage-compensation [15, 21, 27]. Another potential issue concerns the faster turnover rates of tandem paralogs relative to those originating via WGDs [7, 14, 28, 29]. As a consequence, the sampling of tandem paralogs is biased towards young gene duplicates whereas that of WGD paralogs is skewed towards old duplications. As far as we are aware, this bias has not previously been accounted for when inferring patterns of duplicate gene retention.

Here we investigate the impact of duplication mechanism, gene function, and age of duplication in the retention of duplicate genes. Our analyses consider both WGD and tandem duplications, as these are the two most frequently invoked mechanisms to explain how paralogous gene pairs are generated in plant genomes [3, 23, 24, 30]. We have targeted 25 plant species with fully sequenced genomes that include the basal land plants, *Physcomitrella* and *Selaginella*, the basal flowering plant *Amborella*, and as well as 14 flowering plant orders. This diverse array of taxa enables comparisons of taxa with highly contrasting histories of polyploidy, including at least one species with no known WGD in its evolutionary history (*Selaginella*). This is critical, because it allows us to control for potential biases caused by unequal duplicate gene turnover rates. Our focus is on genes annotated as transcription factors, since differential retention of duplicated transcription factors provides the main support for the GBH. We specifically address the following questions: (1) Is the turnover rate of WGD paralogs persistently lower than that of tandem paralogs? (2) Which functional gene categories are consistently overrepresented among WGD and/or tandem paralogs? (3) Does variation in duplicate gene retention depend significantly upon the age of WGD paralogs? and (4) To what extent do our results support for the Gene Balance Hypothesis?

## Results

### Origin and turnover rate of paralogs

**Fig. 1:**
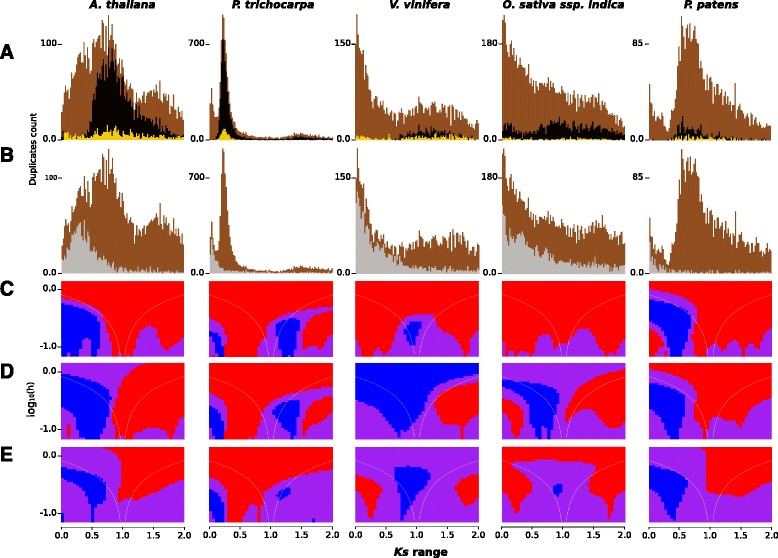
*Ks* age distributions (**a** and **b**) and SiZer maps (**c** to **e**) of five plant species. **a** *Brown bars*, all paralogs (background); *black bars*, WGD-derived paralogs predicted by DAGchainer; *yellow bars*, paralogs annotated as transcription factor activity (GO:0003700). **b** *Brown bars*, background; *gray bars*, tandem-derived paralogs predicted by DAGchainer. SiZer maps for **c** All paralogs; **d** WGD-derived paralogs; **e** Transcription factor paralogs

For each of the 25 study species, we calculated *Ks* time divergence between pairs of paralogs and used a synteny-based approach to categorize members of all gene families as derived from WGD or tandem duplications. Duplicate pairs whose origins were uncertain based on available data were classified as “undefined”. Across the 25 target genomes, the majority of paralogs detected had *Ks* ≤ 2 (Table 1) including 79% of paralogs in *A. thaliana*, 86% in *Glycine max,* and 92% in *Malus domestica*. Paralogs with *Ks* > 2 were excluded from our analyses due to concerns that *Ks* saturation could impair reliable inferences [31]. Most species displayed clear prominent peaks in their *Ks* age histograms, which is illustrated by histograms for five species with contrasting histories of polyploidy (Fig. 1). Histograms for the remaining 20 species are depicted elsewhere (Additional file 1: Figure S1). In the K-S goodness of fit test, all histograms for all species except *Carica* deviated significantly (*P* < 0.05) from the null model of constant duplicate gene birth and death (Additional file 1: Table S1). SiZer maps identified a significantly increasing gradient in the *Ks* age histograms of WGD-derived paralogs of most species, which provides support for polyploid signals being well distinguished from background duplications.

**Table 1:** Distribution of paralogous gene pairs for 25 plant species targeted by this study

Specie	Chr	Initial PCG	Duplicates	Number of duplicates by duplication type			Number of duplicates by *Ks* range				
				WGD	Tandem	Undefined	0 < Ks ≤ 0.5	0.5 < Ks ≤ 1	1 < Ks ≤ 1.5	1.5 < Ks ≤ 2	Ks > 2
*Arabidopsis lyrata*	16	32670	6378	3442	1816	1120	2251	2216	966	945	228
*Arabidopsis thaliana*	10	33602	6194	2740	1232	2222	1657	2407	1183	947	222
*Amborella trichopoda*	26	26460	3322	15	998	2309	1861	427	402	632	137
*Brachypodium distachyon*	10	26678	3573	1025	1768	780	981	1024	835	733	175
*Carica papaya*	18	28072	1915	24	455	1436	603	210	402	700	126
*Citrullus lanatus*	22	23438	2806	385	1015	1406	691	435	751	930	227
*Eucalyptus grandis*	22	36449	11120	390	6424	4306	8106	925	1029	1060	240
*Fragaria vesca*	14	34809	3974	1021	1606	1347	1500	979	684	811	184
*Glycine max*	40	46509	15242	9721	2087	3434	11697	1961	790	794	185
*Helianthus annuus*	34	44144	9925	57	995	8873	4651	2805	1551	918	108
*Lotus japonicus*	12	26818	2682	184	627	1871	1159	774	415	334	51
*Malus domestica*	34	63515	15551	2761	1308	11482	13084	1258	683	526	107
*Manihot esculenta*	36	30800	7134	2530	703	3901	4915	837	716	666	110
*Medicago truncatula*	16	57587	5098	1083	2419	1596	2902	1262	543	391	115
*Oryza sativa ssp. indica*	24	48788	8349	1957	2869	3523	3361	2169	1665	1154	317
*Oryza sativa ssp. japonica*	24	59430	5559	1482	2173	1904	1928	1584	1233	814	183
*Physcomitrella patens*	54	36137	3769	306	202	3261	637	1848	883	401	99
*Populus trichocarpa*	36	41521	9721	5609	1988	2124	7572	738	704	707	147
*Ricinus communis*	20	31221	2558	155	614	1789	628	435	683	812	176
*Sorghum bicolor*	20	34686	4267	1048	1698	1521	1468	1061	993	745	186
*Solanum lycopersicum*	24	34432	7100	1234	2561	3305	3184	2287	872	757	209
*Selaginella moellendorffii*	16–27	22285	1885	351	608	926	1457	129	102	197	66
*Theobroma cacao*	20	46269	3488	722	1553	1213	1199	601	822	866	201
*Vitis vinifera*	38	26644	4536	528	1935	2073	1918	852	1042	724	128
*Zea mays*	20	39597	6336	590	1396	4350	3792	1095	813	636	153

*Chr* Number of Chromosomes, *Initial PCG* Initial number of Protein-coding gene sequences

The *Ks* age histograms of WGD-derived paralogs (Fig. 1a, depicted in black) were clearly distinct from those of the tandem-derived paralogs (Fig. 1b, depicted in gray). While tandem histograms exhibited a descending slope (similar to a half-parabola) for most of the species, WGD-derived paralog histogams had peaks that overlapped with peaks from histograms of all paralogs (Fig. 1a, depicted in brown). SiZer maps also confirmed the presence of peaks for WGD-derived paralogs histograms (Fig. 1d).

Because of our focus on transcription factor paralogs, their *Ks* age histograms are shown (Fig. 1 and Additional file 1: Figure S1; depicted in yellow) along with the *Ks* histograms of WGD- and tandem-derived paralogs. The SiZer maps (Fig. 1e) showed increasing gradients for transcription factor paralogs that overlapped with the slopes of WGD-derived paralogs.

### Biased retention of paralogs after large- and small-scale duplications

**Fig. 2:**
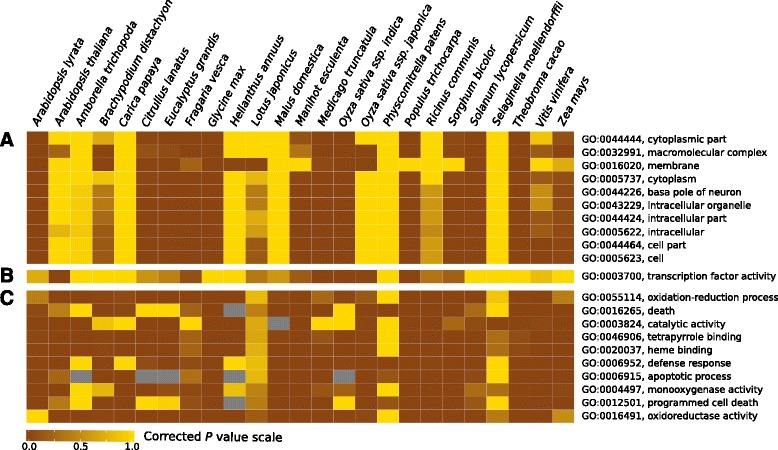
Heat maps of GO categories across 25 plant species. **a** The 10 most frequent GO categories overrepresented among WGD-derived paralogs. **b** Transcription factor activity category (GO:0003700) enrichment analysis for WGD-derived paralogs. **c** The 10 most frequent GO categories overrepresented among tandem-derived paralogs. Color gradient represents the Corrected *P* value calculated by the ErmineJ software: *brown colors*, significant over-representation (*P < 0.05*); *yellow colors*, reduced or non-significant enrichment; and *gray color*, no enrichment

To assess the universality of the GBH across land plants, we identified the most strongly overrepresented GO functional categories in both predicted WGD- and tandem-derived paralogs in these 25 genomes. We found that WGD- and tandem-derived paralogs did not share the top 10 most frequently overrepresented GO categories (Fig. 2a and c). While the most overrepresented categories of WGD-derived paralogs fell under macromolecular complexes (GO:0032991), internal to cell (GO:0005622), and cytoplasm (GO:0005737) functional GO categories; those of tandem-derived paralogs grouped into programmed cell death (GO:0012501), defense response (GO:0006952), and apoptotic process (GO:0006915) GO categories.

In six species, WGD-derived paralogs were not enriched for the overrepresented GO categories found in the remaining plant species. Five of them—*Cariaca*, *Ricinus, Populus*, *Selaginella*, and *Physcomitrella—*have few WGD-derived paralogs predicted by DAGchainer (Table 1), consistent with possible under-estimation or misidentification of WGD-derived paralogs in these species (see Discussion below). For another five taxa—*A. thaliana*, *Medicago,* both *Oryza* subspecies*,* and *Populus—*WGD-derived transcription factor paralogs were overrepresented (Fig. 2b)*.* Surprisingly, WGD-derived transcription factor paralogs were not significantly overrepresented in *Arabidopsis lyrata*, which shares the same WGD events as *A. thaliana*, although there was a trend in the expected direction.

Unexpectedly, transcription factor activity (GO:0003700) WGD-derived paralogs were not significantly overrepresented in 20 plant species, ten of which exhibit evidence of recent WGDs in their evolutionary history, with a significantly increasing gradient in SiZer (Fig. 1 and Additional file 1: Figure S1) within *Ks* range < 1 (and consistent with previous reports—see below). Finally, results from our analyses of tandem duplications showed tandem-derived transcription factor paralogs were significantly underrepresented across the 25 focal genomes.

### Biased retention toward ancient transcription factors

**Fig. 3:**
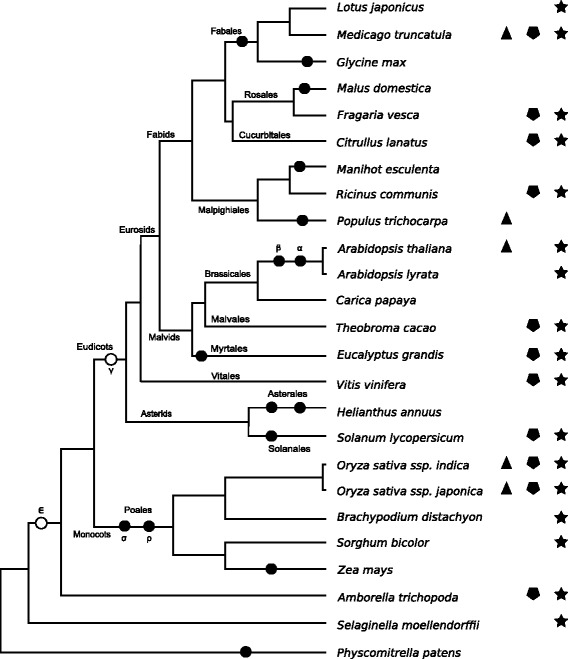
Phylogenetic distribution of transcription factor retention biases among 25 plant species. The phylogenetic tree was adapted from PLAZA 3.0. Symbol code: *Black circles* on the tree branches, all known WGD events we also identified in this study; *Open circles*, suggested ancient WGD events we did not examine; *triangles*, species with WGD-derived transcription factor paralogs significantly overrepresented; *pentagons* and *stars*, species with transcription factor paralogs significantly overrepresented in range 1.5 < *Ks* ≤ 2 and range 1 < *Ks* ≤ 2, respectively

We analyzed the biased retention of transcription factor paralogs based on *Ks* time divergence as opposed to mechanism of duplication. This was accomplished by mapping known WGD events onto a phylogeny for the 25 species targeted by this study (Fig. 3, Additional file 1: Table S2).

In general, transcription factor (GO:003700) paralogs tend to be overrepresented amongst ancient (*Ks* > 1) duplication regardless of mechanism of duplication (Fig. 3). Eleven of the 25 focal species exhibited significant enrichment at *Ks* range > 1.5 (Fig. 3, pentagons), but no such retention bias at lower *Ks* ranges (≤1.5). When we compared transcription factor paralog enrichment at *Ks* > 1.0 versus < 1.0, 17 species showed significant enrichment for the older transcription factor paralogs (Fig. 3, stars). For four of these, *A. thaliana*, *Medicago*, and the two *Oryza* subspecies, the overrepresented transcription factors originated from WGD events (Fig. 2b). However, for the remaining 13 species, the ancient paralogs are not obviously associated with a WGD event. Although *A. thaliana, Oryza sativa ssp. indica,* and *Solanum* exhibited significant signals of polyploidy in the *Ks* range < 1 (Fig. 1; Additional file 1: Figure S1), their transcription factor paralogs were only significantly overrepresented in the *Ks* range > 1 (Fig. 3f).

In genomes of only four taxa (*Carica*, *Malus*, *Manihot,* and *Populus*) were recent transcription factor paralogs overrepresented, and only for *Populus* were WGD-derived transcription factor paralogs significantly overrepresented (Fig. 2b).

In addition to analyzing the retention of transcription factor paralogs, we submitted our data to enrichment analysis aiming to find additional GO categories that could have experienced biased retention patterns. A number of GO categories, including those involved in transcription, regulation, transport, and response to stimulus were frequently overrepresented among ancient paralogs (*Ks* > 1) and not exclusively associated to WGDs (Additional file 1: Figure S2). While three of these functional GO categories—cell periphery (GO:0071944), plasma membrane (GO:0005886), and response to abiotic stimulus (GO:0009628)—were overrepresented among WGD-derived paralogs; two categories—response to stimulus (GO:0050896) and catalytic activity (GO:0003824)—were overrepresented among tandem-derived paralogs.

## Discussion

### Tandem paralogs have faster turnover rate

Our synteny-based approach identified pairs of WGD-derived genes similar to those that have been reported in previous studies. In *A. thaliana*, for example, circa 80% of the 2740 duplicate gene pairs we classified as WGD-derived are in common with the list of polyploidy-derived paralogs published by [23]. Differences among studies may be due to new gene annotation tools that recently became available. In some instances, the number of paralogs predicted as having their origin in WGD events can be underestimated due to widespread genomic changes (e.g., gene loss and/or chromosomal rearrangements) after polyploidization events [19]. Such processes are particularly problematic for ancient polyploidization events, which may explain the low number of WGD paralogs we predicted in the basal plants, *Amborella* and *Physcomitrella*, as well as for *Lotus*, *Carica*, and *Ricinus* (Table 1). On the other hand, our approach indicates the presence of a small number WGD-derived paralogs in *Selaginella,* which is not thought to have a WGD in its evolutionary history (Table 1). This result could be evidence for *Selaginella* as ancient polyploidy. Alternatively, it suggests that *Selaginella* has had an ancient large segmental duplication or some fraction of the identified WGD derived paralogs are false positives. However, we selected WGD pairs using a syteny based approach, which is the most conservative method presently available.

Tandem paralogs were similarly identified based on the genomic coordinates of genes. In *Eucalyptus*, 32% of its 36,449 protein-coding genes originated via tandem events, which is the largest proportion of tandem-derived paralogs amongst the 25 plant species we investigated. *Physcomitrella* exhibited the smallest proportion (~1%) of tandem-derived paralogs. These findings are very similar to those previously reported for *Eucalyptus* [32] and *Physcomitrella* [33], respectively.

We identified peaks in the *Ks* age histograms; based on SiZer maps, these peaks likely result from WGDs (Fig. 1 and Additional file 1: Figure S1). Previous studies have also identified these WGD events using data that span across several families [34], or from a given plant species [9, 10, 32]. In the *Ks* histogram of *A. thaliana*, for example, there were two prominent peaks (Fig. 1), which coincided with the α and β polyploid events reported by early investigations [34–36]. In our analysis, the tail of the most recent duplication masked the second peak; thus, a single, significantly increasing slope was identified by SiZer. In *A. lyrata,* SiZer identified two significant peaks as expected given the recent history of polyploidy in *Arabidopsis* [36].

Differences in the *Ks* age histograms from WGD- and tandem-derived paralogs indicates that the turnover rate of tandem paralogs is faster than that of WGD paralogs, as previously suggested by others [7, 14, 29, 33]. The pattern we uncovered suggests lower turnover rates of transcription factor paralogs than those observed for tandem paralogs. Furthermore, it appears that the origin and biased retention of transcription factor paralogs are not restricted to large-scale duplication events.

### Patterns of transcription factor retention following WGDs

Consistent with the expectations of the GBH, WGD- and tandem-derived paralogs did not share the top 10 most frequently overrepresented GO categories. Six species*—Malus, Cariaca*, *Ricinus, Populus*, *Selaginella*, and *Physcomitrella—*were exceptions and did not share the most frequent GO categories, which is consistent with the possible under-estimation or misidentification of WGD-derived paralogs in these species. In *Malus*, for example, the GO categories that were overrepresented include: plasma membrane (GO:0005886), response to abiotic stimulus (GO:0009628), response to biotic stimulus (GO:0009607), and response to endogenous stimulus (GO:0009719). Analyses of an EST library of *Malus domestica* also found that these categories were overrepresented [37]. Consistent with the GBH, we did not found tandem-derived transcription factor paralogs overrepresented in any of 25 focal genomes.

Other findings were inconsistent with the predictions of the GBH. In plants, the genome of *A. thaliana* has been frequently used to support dosage-constraints of transcription factors [23, 24]. Unexpectedly, our findings reveal transcription factor activity (GO:0003700) WGD-derived paralogs to be significantly overrepresented in only five plant taxa—*A. thaliana*, *Medicago,* the two *Oryza* subspecies*,* and *Populus*. Ten of the 20 remaining study species exhibited evidence for recent WGDs. Other studies have also reported a downward bias in the retention of transcription factor paralogs following WGD events. In Compositae paleologs, for example, it has been observed that genes involved with structural components or cellular organization were significantly overrepresented; whereas transcription factors were significantly underrepresented [10]. These authors argued that patterns of intrinsic selection on different gene categories may vary across higher taxonomic categories. The fate of paralogs originated by either WGD or small-scale events would depend on intrinsic properties, such as gene function and the environment in which the new polyploid was born [21].

### Age of duplications contribute to biased gene retention

Regardless the mechanism of duplication, we showed that ancient paralogs of transcription factors were preferentially retained over paralogs of more recent origin. In agreement to our findings, a previous study in *A. thaliana* reported that genes involved in transcriptional regulation showed greater retention after the later (β) genome duplication than after the youngest (α) duplication [24]. Likewise, transcription factors not directly associated with WGDs were overrepresented among genes of ancient origin in *A. thaliana* [13]. Again, our results indicate that out of 25 plant species with very different histories of polyploidy, such as *A. thaliana* which has two recent WGD events [35] and *Vitis* which has no known recent WGD events [9], 17 share this pattern of biased retention of ancient transcription factor paralogs. Although transcription factor paralogs with recent origin were over-represented in four species (*Carica*, *Malus*, *Manihot,* and *Populus*), we could only clearly determine that those of *Populus* were WGD-derived paralogs. The over-representation of young (*Ks* < 0.5) transcription factor paralogs in *Carica* is intriguing, given that no WGD events likely took place in its recent evolutionary history [38] and that DAGchainer only predicted tandem-derived transcription factor paralogs for *Carica* within the *Ks* range ≤ 1.0 (Additional file 1: Table S2). Given that *Carica* lacks recent WGD events [38] and we did not identified transcription factors paralogs originated from WGD events within *Ks* < 1, the many transcription factor paralogs of *Carica* appear to derive from small-scale duplications within its genome.

Our findings differ from a recent study of core gene families in 37 angiosperm genomes [13], which reported remarkable consistency in the rate at which genes return to a single copy state, as well as in the gene families that are retained as multi-copy. The findings were related to differences in gene function and the authors concluded that similar selection pressures within and between lineages are largely responsible for the non-random patterns observed, at least for core genes [13]. The apparent differences between the two studies derive partly from the fact that core gene families represent a fairly small fraction (13%) of all gene families and that single copy genes in were included their analysis, which drive many of the reported patterns. In contrast, we restricted our analyses to duplicate genes.

## Conclusions

Our analyses imply that both the fixation and retention of duplicated genes are context-dependent events. Thus, while the mechanism of duplication is clearly important, so are the characteristics of the particular lineage in which the duplication arises, as well as timing of duplication. Although our results show that many transcription factor paralogs do indeed derive from large-scale duplication events, this is not conclusive evidence for the GBH. Observations seemingly inconsistent with the GBH include, for example, the preferential retention of transcription factor paralogs in taxa with no apparent history of polyploidy or following tandem duplications in *Carica*, as well as the absence of such retention biases following some recent WGDs (e.g. *Glycine, Helianthus,* and *Zea*). Nonetheless, the most important observation in this paper—the strong bias toward ancient transcription factor duplicates seen in most plant genomes—may be interpreted in a manner consistent with the GBH. Possibly, all plant lineages are the product of multiple ancient WGDs, the earliest of which are no longer detectable. Under the GBH, the duplicates from the first polyploidization would be most likely to be retained to maintain optimal stoichiometry among gene products. The number of paralogs is expected to grow rapidly with each polyploidization event. With so many paralogs, changes in the amount of the gene product might be tolerated and a copy of the gene can be lost or diverge. This could lead to the pattern we see—biased retention toward ancient transcription factor paralogs—and also might account for the weaker signal we see among recent transcription factor paralogs. It even could account, in part, for the greater tolerance of recent tandem transcription paralogs seen in *Carica*.

## Methods

### Data collection and selection of paralogs

Full genome annotations, protein-gene codes, DNA sequences, gene families, and Gene Ontology (GO) annotations from the 25 focal species were retrieved from PLAZA 2.5 and 3.0 Dicots [39], with the exception of sunflower (*Helianthus annuus*), as detailed in Additional file 1: Table S3. Protein-gene code files with alternative transcripts removed were used to identify paralogous gene pairs using BLASTp all-against-all, with an *e*-value cutoff of e^−20^, with a minimum 50% identity, alignment length > 300 bp, number of mismatches < 550, and number of gap opens < 30. Self hits were removed and only paralogous gene pairs with both copies belonging to the same gene family were maintained for further analysis. For the selection of paralog pairs for *H. annuus*, CDS sequences and BLASTn all-against-all were used based on HA412.v1.1 version of the genome (http://www.sunflowergenome.org/).

### Determining paralog duplication mechanism

The DAGchainer software package [40] was used to predict the mechanism of by which paralogs originated based on their genomic coordinates. WGD-derived paralog pairs were predicted by running DAGchainer to find syntenic/collinear regions among chromosomes, in the same species, using default parameters and ignoring tandem duplication alignments (-s and -I options). Tandem-derived paralogs were predicted by using the accessory segmental duplication tool, also made available by DAGchainer, to find collinear sets of homologous genes, with the ‘max intervening genes value’ set to 10. All the other paralog pairs, not predicted as WGD or tandem-derived, were marked as undefined (UD), as these paralogs may have been originated by either large- or small-scale duplications.

### Age of duplication events

We calculated relative divergence times for each paralog pair in terms of synonymous substitutions per synonymous site (*Ks*). First, we aligned the nucleotide sequences of gene pairs using TranslatorX [41], based on protein alignments performed by MUSCLE v3.8.31 [42]. Divergence times (*Ks*) were calculated with the *yn00* software from the PAML v4.1 package [43]. This method assumes the F3x4 codon frequency model and accounts for transition/transversion rate bias and codon usage bias, which is an approximation of the maximum likelihood method recommended for pairwise comparisons in the manual of PAML. Because of issues associated with *Ks* saturation and stochasticity [31], only paralogs with *Ks* ≤ 2 and Standard Error (SE) < 0.5 were used in further analyses.

Custom python scripts were used to parse the BLAST all-against-all output in order to identify the closest paralog gene pairs. First, self hits were removed. Then, paralogs were organized into a single gene list and then used to select the corresponding paralog pair(s) for each of these genes based on the following three rules: (I) if a single gene was predicted as WGD-derived by DAGchainer, keep the duplicate pair with the lowest *Ks* value, while still allowing pairing with tandem-derived or undefined genes; (II) if not predicted as WGD-derived, but predicted as tandem-derived, keep the gene pair with the lowest *Ks* value; and (III) if the single gene was not predicted as WGD- or tandem-derived, keep the undefined paralog pair with the lowest *Ks* value.

### GO annotation and over-representation analysis

Functional Gene ontology GO terms (categories) were determined for each gene and paralog pair and then evaluated for enrichment by the ErmineJ v3.0.2 software [44]. All the three GO domains (Biological Process, Molecular Function and Cellular Component) were included in the Over-Representation Analysis (ORA), with a minimum gene set size equal to 10 and the Best Scoring gene replicate treatment. Eight different groups of paralogs were analyzed: WGD-derived, tandem-derived, and paralogs representing the following *Ks* ranges: (A) 0 < *Ks* ≤ 0.5, (B) 0.5 < *Ks* ≤ 1, (C) 1 < *Ks* ≤ 1.5, (D) 1.5 < *Ks* ≤ 2, (E) 0 < *Ks* ≤ 1 and (F) 1 < *Ks* ≤ 2. The GO categories were considered overrepresented if Corrected *P* < 0.05, as calculated by the ErmineJ software.

### Statistics

#### K-S goodness of fit test

The Kolmogorov-Smirnov test [45] was used to evaluate if the age distribution (*Ks*) of all duplicates (background) deviated significantly (*P* < 0.05) from a simulated null hypothesis of constant duplicate gene birth and death.

### SiZer maps: identifying significant peaks in Ks histograms

Significant peaks in the *Ks* histograms were found by SiZer [46] implemented on R software, with the following command line: SiZer.1 < − SiZer(x, y, h = c(.05,5), degree = 1, derv = 1). A SiZer map is a way of examining when the p-th derivative of a scatterplot-smoother is significantly negative, possibly zero or significantly positive across a range of smoothing bandwidths. In a SiZer map, blue indicates a significantly increasing gradient, red is a significantly decreasing gradient, purple is a non-significant gradient and gray indicates that data are too sparse for reliable estimation.

## Additional file

Additional file 1: Figure S1.
*Ks* age distributions of 20 species and correspondent maps. In (A) brown bars represent all duplicates (background), black and yellow represent the WGD-derived duplicates predicted by DAGchainer and transcription factors activity (GO:0003700) duplicates, respectively. (B) Brown bars represent the background and gray the tandem-derived duplicates. (C) SiZer maps of background. (D) SiZer maps of WGD-derived duplicates. (E) SiZer maps of transcription factors duplicates. **Figure S2.** Gene Ontology (GO) categories overrepresented in duplicates with (a) 1.5 < Ks ≤ 2, and (b) 1 < Ks ≤ 2 in nine or more of the 25 plant species analyzed in this study. **Table S1.** Kolmogorov-Smirnov test for *Ks* age distributions of all duplicates of the 25 plant species used in this study. **Table S2.** Number of duplicates annotated as transcription factor (TF) activity (GO:0003700) for 25 plant species, displayed by duplication type (predicted by the DAGchainer software) and grouped by *Ks* equivalent ages ranges. **Table S3.** Detailing the data source and abbreviation of the 25 plant species used in this study. (PDF 2652 kb)

## References

[1] Ohno S (1970). Evolution by Gene Duplication.

[2] Wendel JF (2000). Genome evolution in polyploids. Plant Mol Biol.

[3] Kondrashov FA, Rogozin IB, Wolf YI, Koonin EV (2002). Selection in the evolution of gene duplications. Genome Biol.

[4] Zhang J (2003). Evolution by gene duplication: an update. Trends Ecol Evol.

[5] Wood TE, Takebayashi N, Barker MS, Mayrose I, Greenspoon PB, Rieseberg LH (2009). The frequency of polyploid speciation in vascular plants. Proc Natl Acad Sci U S A.

[6] Mayrose I, Zhan SH, Rothfels CJ, Magnuson-Ford K, Barker MS, Rieseberg LH (2011). Recently formed polyploid plants diversify at lower rates. Science (80- ).

[7] Blanc G, Wolfe KH (2004). Functional Divergence of Duplicated Genes Formed by Polyploidy during Arabidopsis Evolution. Plant Cell.

[8] Shoemaker RC, Schlueter J, Doyle JJ (2006). Paleopolyploidy and gene duplication in soybean and other legumes. Curr Opin Plant Biol.

[9] Jaillon O, Aury J-M, Noel B, Policriti A, Clepet C, Casagrande A (2007). The grapevine genome sequence suggests ancestral hexaploidization in major angiosperm phyla. Nature.

[10] Barker MSS, Kane NCC, Matvienko M, Kozik A, Michelmore RW, Knapp SJJ (2008). Multiple paleopolyploidizations during the evolution of the Compositae reveal parallel patterns of duplicate gene retention after millions of years. Mol Biol Evol.

[11] Jiao Y, Wicket N, Ayyampalayam S, Chanderbali A (2011). Ancestral polyploidy in seed plants and angiosperms. Nature.

[12] Li Z, Baniaga AE, Sessa EB, Scascitelli M, Graham SW, Rieseberg LH (2015). Early genome duplications in conifers and other seed plants. Sci Adv.

[13] Li Z, Defoort J, Tasdighian S, Maere S, Van de Peer Y, De Smet R (2016). Gene Duplicability of Core Genes Is Highly Consistent across All Angiosperms. Plant Cell.

[14] Lynch M, Conery JS (2000). The Evolutionary Fate and Consequences of Duplicate Genes. Science (80- ).

[15] Edger PP, Pires JC (2009). Gene and genome duplications: the impact of dosage-sensitivity on the fate of nuclear genes. Chromosom Res.

[16] Edger PP, Heidel-Fischer HM, Bekaert M, Rota J, Glöckner G, Platts AE (2015). The butterfly plant arms-race escalated by gene and genome duplications. Proc Natl Acad Sci U S A.

[17] Conant GC, Birchler JA, Pires JC (2014). Dosage, duplication, and diploidization: clarifying the interplay of multiple models for duplicate gene evolution over time. Curr Opin Plant Biol.

[18] Birchler J, Bhadra U, Bhadra MP, Auger DL (2001). Dosage-dependent gene regulation in multicellular eukaryotes: implications for dosage compensation, aneuploid syndromes, and quantitative traits. Dev Biol.

[19] Freeling M (2009). Bias in Plant Gene Content Following Different Sorts of Duplication: Tandem, Whole-Genome, Segmental, or by Transposition. Annu Rev Plant Biol.

[20] Kondrashov FA, Koonin EV (2004). A common framework for understanding the origin of genetic dominance and evolutionary fates of gene duplications. Trends Genet.

[21] Hudson CM, Puckett EE, Bekaert M, Pires JC, Conant GC (2011). Selection for higher gene copy number after different types of plant gene duplications. Genome Biol Evol.

[22] Veitia RA (2002). Exploring the etiology of haploinsufficiency. Bioessays.

[23] Blanc G, Wolfe KH (2004). Widespread Paleopolyploidy in Model Plant Species Inferred from Age Distributions of Duplicate Genes. Plant Cell.

[24] Maere S, De Bodt S, Raes J, Casneuf T, Van Montagu M, Kuiper M (2005). Modeling gene and genome duplications in eukaryotes. Proc Natl Acad Sci U S A.

[25] Yu J, Wang J, Lin W, Li S, Li H, Zhou J (2005). The Genomes of Oryza sativa: A History of Duplications. PLoS Biol.

[26] Rodgers-Melnick E, Mane SP, Dharmawardhana P, Slavov GT, Crasta OR, Strauss SH (2012). Contrasting patterns of evolution following whole genome versus tandem duplication events in Populus. Genome Res.

[27] Crow KD, Wagner GP (2006). What is the role of genome duplication in the evolution of complexity and diversity?. Mol Biol Evol.

[28] Hanada K, Zou C, Lehti-Shiu MD, Shinozaki K, Shiu S-H (2008). Importance of Lineage-Specific Expansion of Plant Tandem Duplicates in the Adaptive Response to Environmental Stimuli. Plant Physiol.

[29] Wang Y (2013). Locally duplicated ohnologs evolve faster than nonlocally duplicated ohnologs in Arabidopsis and rice. Genome Biol Evol.

[30] Thomas BC, Pedersen B, Freeling M (2006). Following tetraploidy in an Arabidopsis ancestor, genes were removed preferentially from one homeolog leaving clusters enriched in dose-sensitive genes. Genome Res.

[31] Vanneste K, Van De Peer Y, Maere S (2013). Inference of genome duplications from age distributions revisited. Mol Biol Evol.

[32] Myburg AA, Grattapaglia D, Tuskan GA, Hellsten U, Hayes RD, Grimwood J (2014). The genome of Eucalyptus grandis. Nature.

[33] Rensing SA, Lang D, Zimmer AD, Terry A, Salamov A, Shapiro H (2008). The Physcomitrella genome reveals evolutionary insights into the conquest of land by plants. Science (80- ).

[34] Vanneste K, Baele G, Maere S, Van De Peer Y (2014). Analysis of 41 plant genomes supports a wave of successful genome duplications in association with the Cretaceous-Paleogene boundary. Genome Res.

[35] Simillion C, Vandepoele K, Van Montagu MCE, Zabeau M, Van De Peer Y (2002). The hidden duplication past of Arabidopsis thaliana. PNAS.

[36] Bowers JE, Chapman BA, Rong J, Paterson AH (2003). Unrevealing angiosperm genome evolution by phylogenetic analysis of chromosomal duplication events. Nature.

[37] Sanzol J. Dating and functional characterization of duplicated genes in the apple (Malus domestica Borkh.) by analyzing EST data. BMC Plant Biol. 2010;10:87. 10.1186/1471-2229-10-87 PMC3095355 20470375

[38] Ming R, Hou S, Feng Y, Yu Q, Dionne-Laporte A, Saw JH (2008). The draft genome of the transgenic tropical fruit tree papaya (*Carica papaya* Linnaeus). Nature.

[39] Proost S, Van Bel M, Vaneechoutte D, Van de Peer Y, Inzé D, Mueller-Roeber B (2015). PLAZA 3.0: an access point for plant comparative genomics. Nucleic Acids Res.

[40] Haas BJ, Delcher AL, Wortman JR, Salzberg SL (2004). DAGchainer: a tool for mining segmental genome duplications and synteny. Bioinformatics.

[41] Abascal F, Zardoya R, Telford MJ (2010). TranslatorX: multiple alignment of nucleotide sequences guided by amino acid translations. Nucleic Acids Res.

[42] Edgar RC (2004). MUSCLE: multiple sequence alignment with high accuracy and high throughput. Nucleic Acids Res.

[43] Yang Z (2007). PAML 4: phylogenetic analysis by maximum likelihood. Mol Biol Evol.

[44] Gillis J, Mistry M, Pavlidis P (2010). Gene function analysis in complex data sets using ErmineJ. Nat Protoc.

[45] Cui L, Wall PK, Leebens-Mack JH, Lindsay BG, Soltis DE, Doyle JJ (2006). Widespread genome duplications throughout the history of flowering plants. Genome Res.

[46] Chaudhuri P, Marron JS (2000). Scale space view of curve estimation. Ann Stat.

