# Supplementary material for: Both mechanism and age of duplications contribute to biased gene retention patterns in plants
Source: BMC Genomics. 2017 Jan 6;18:46. doi: 10.1186/s12864-016-3423-6 (PMC5219802; doi:10.1186/s12864-016-3423-6)
Supplement: Additional file 1: Figure S1. — Ks age distributions of 20 species and correspondent maps. In (A) brown bars represent all duplicates (background), black and yellow represent the WGD-derived duplicates predicted by DAGchainer and transcription factors activity (GO:0003700) duplicates, respectively. (B) Brown bars represent the background and gray the tandem-derived duplicates. (C) SiZer maps of background. (D) SiZer maps of WGD-derived duplicates. (E) SiZer maps of transcription factors duplicates. Figure S2. Gene Ontology (GO) categories overrepresented in duplicates with (a) 1.5 < Ks ≤ 2, and (b) 1 < Ks ≤ 2 in nine or more of the 25 plant species analyzed in this study. Table S1. Kolmogorov-Smirnov test for Ks age distributions of all duplicates of the 25 plant species used in this study. Table S2. Number of duplicates annotated as transcription factor (TF) activity (GO:0003700) for 25 plant species, displayed by duplication type (predicted by the DAGchainer software) and grouped by Ks equivalent ages ranges. Table S3. Detailing the data source and abbreviation of the 25 plant species used in this study. (PDF 2652 kb) [file 12864_2016_3423_MOESM1_ESM.pdf]

Additional Files

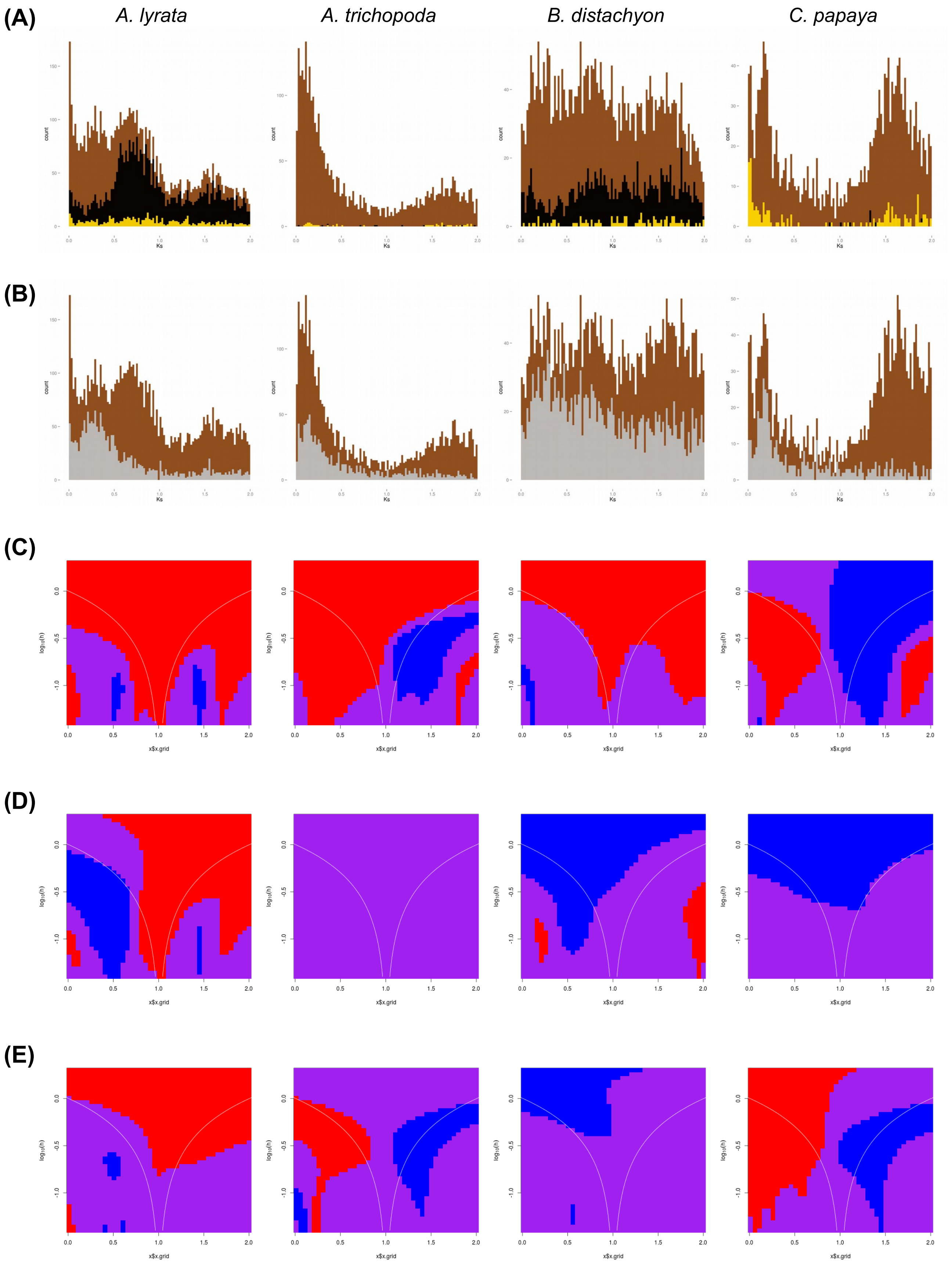
(A)
A. lyrata
A. trichopoda
B. distachyon
C. papaya
(B)
(C)
(D)
(E)
count
Ks
$\log_{10}(h)$
x$x.grid

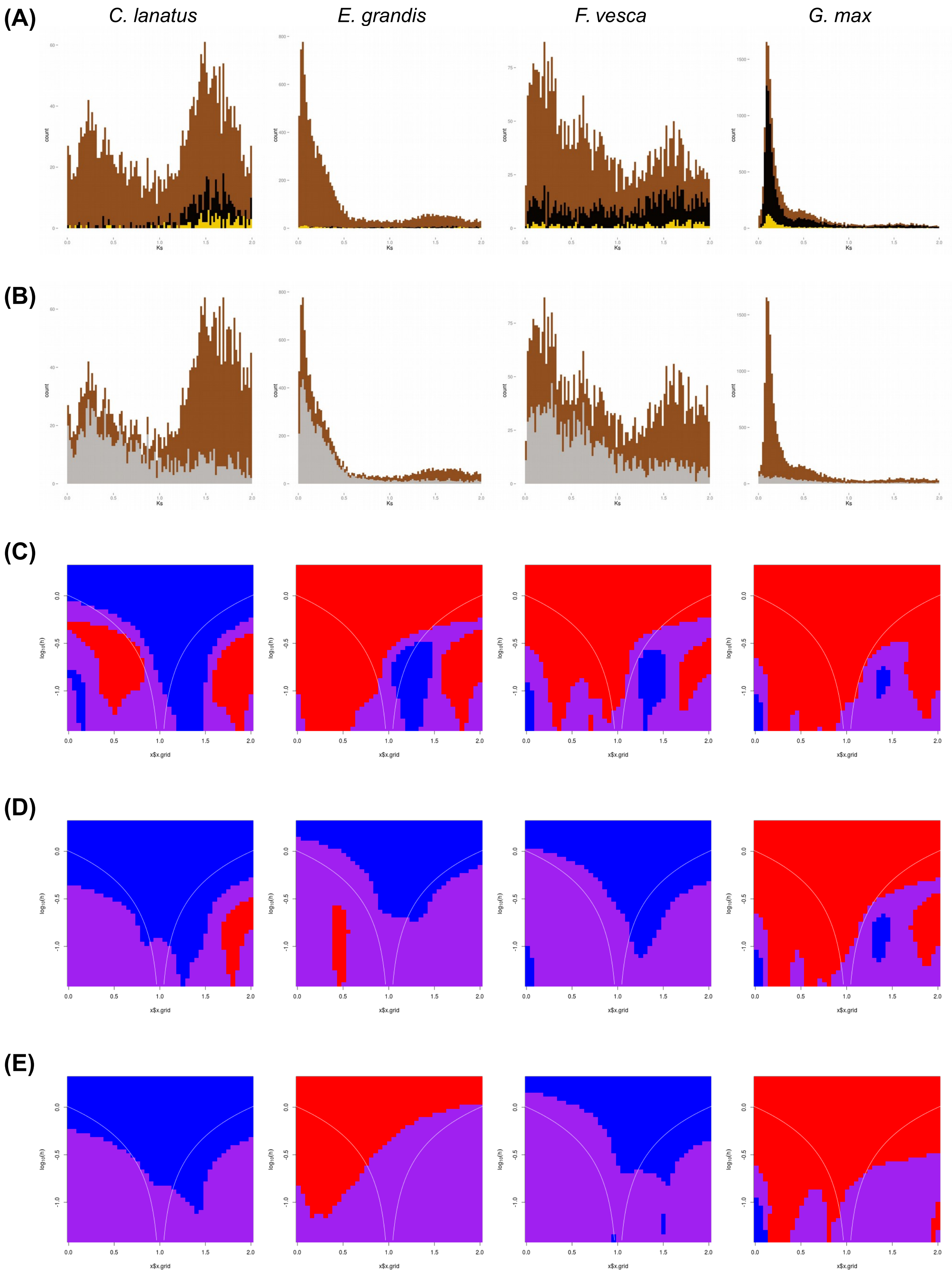
(A)
C. lanatus
E. grandis
F. vesca
G. max
count
Ks
(B)
count
Ks
(C)
log10(h)
x$x.grid
(D)
log10(h)
x$x.grid
(E)
log10(h)
x$x.grid

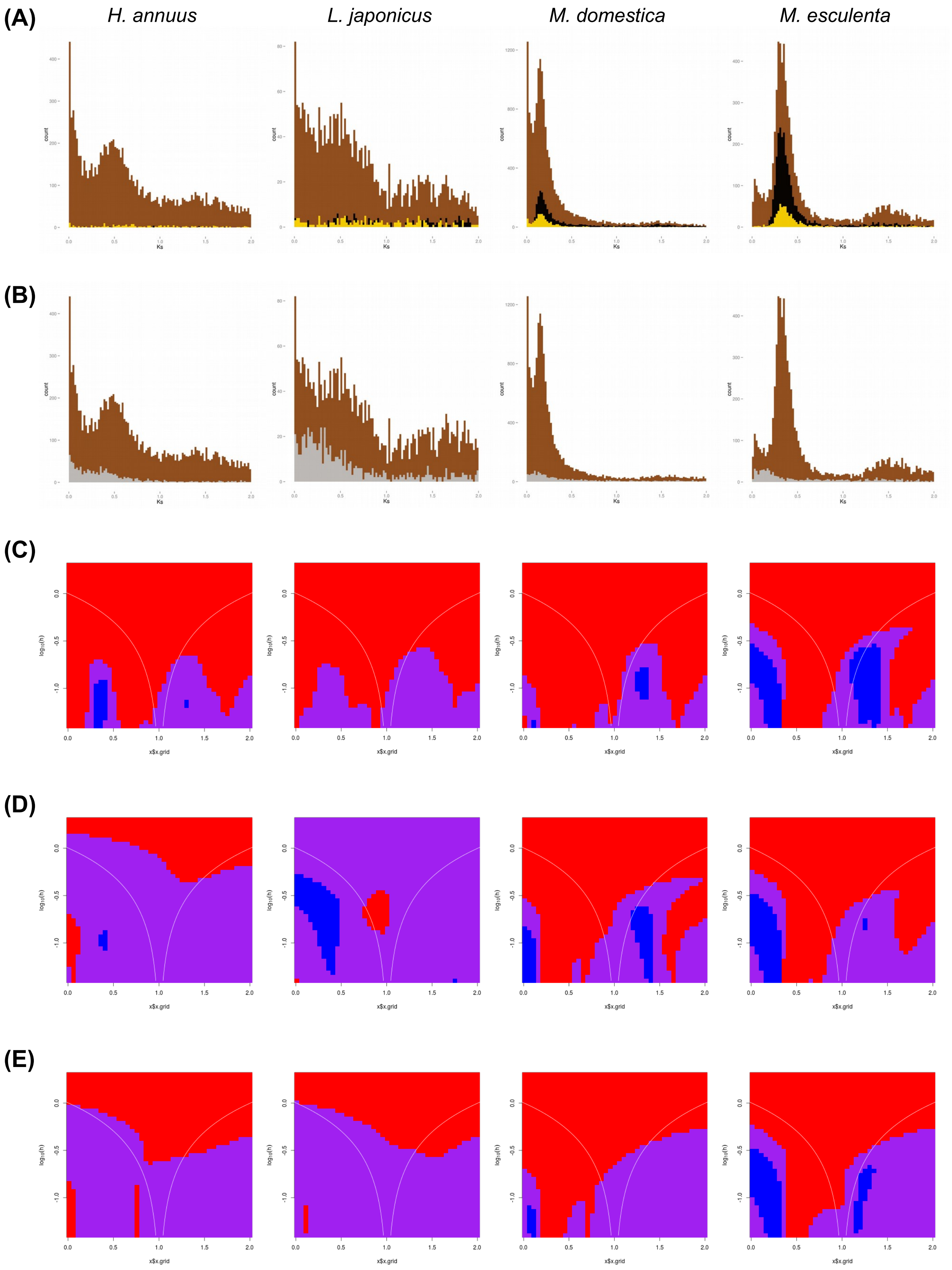
(A)
H. annuus
L. japonicus
M. domestica
M. esculenta
count
Ks
(B)
count
Ks
(C)
log10(h)
x$x.grid
(D)
log10(h)
x$x.grid
(E)
log10(h)
x$x.grid

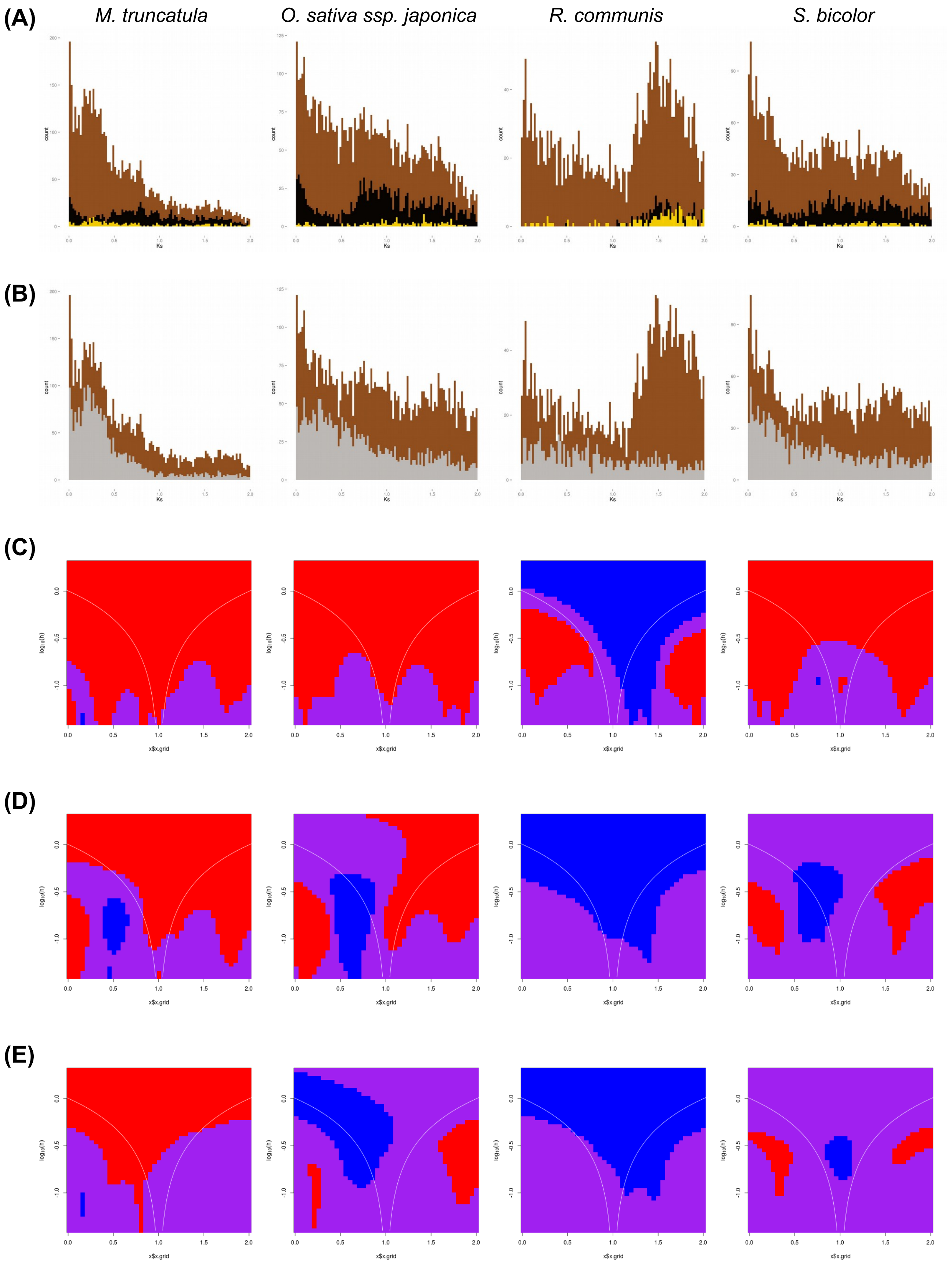(A)
M. truncatula
O. sativa ssp. japonica
R. communis
S. bicolor
(B)
(C)
(D)
(E)
count
Ks
log10(h)
x$x.grid

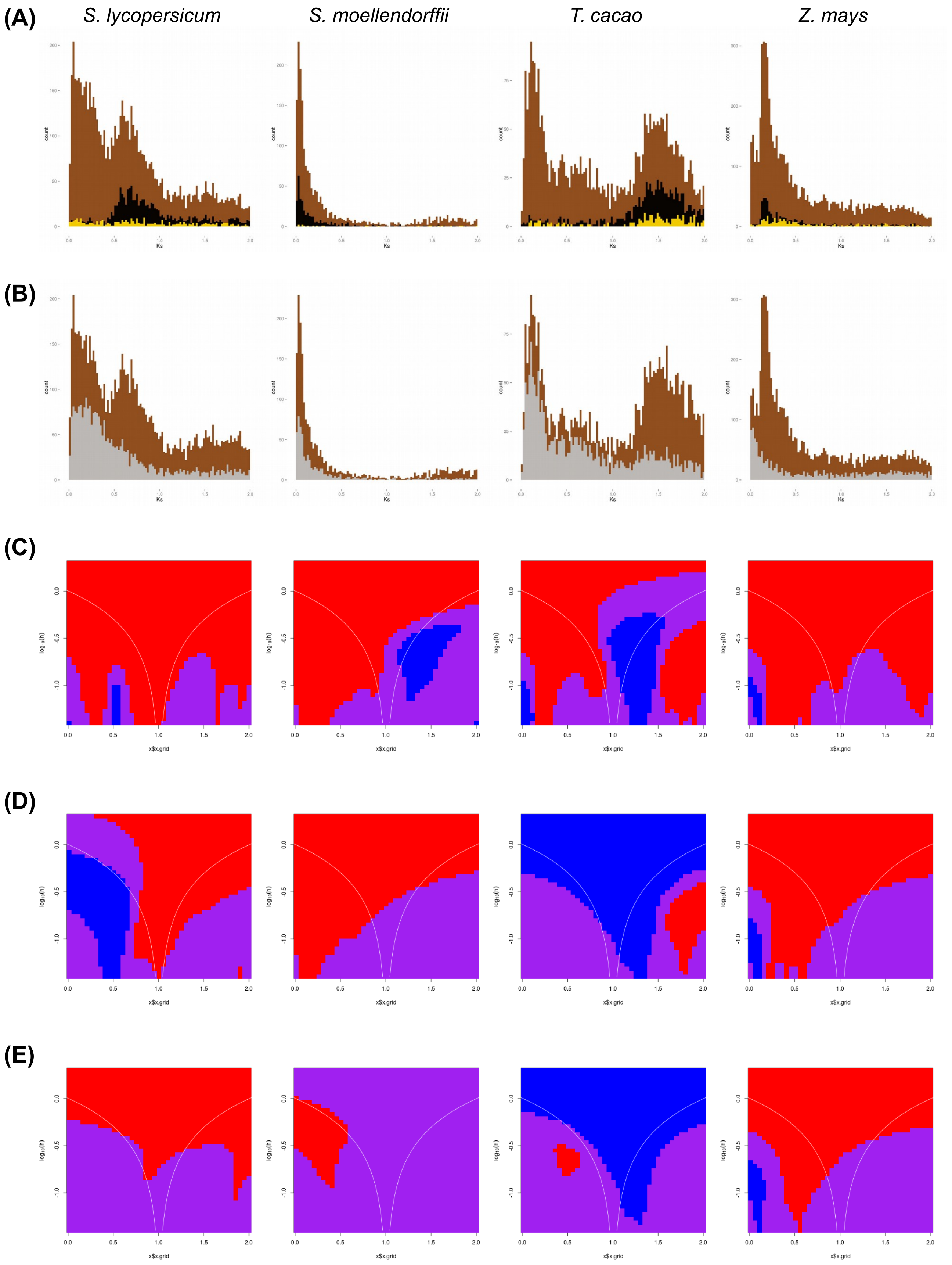

Additional Figure 1

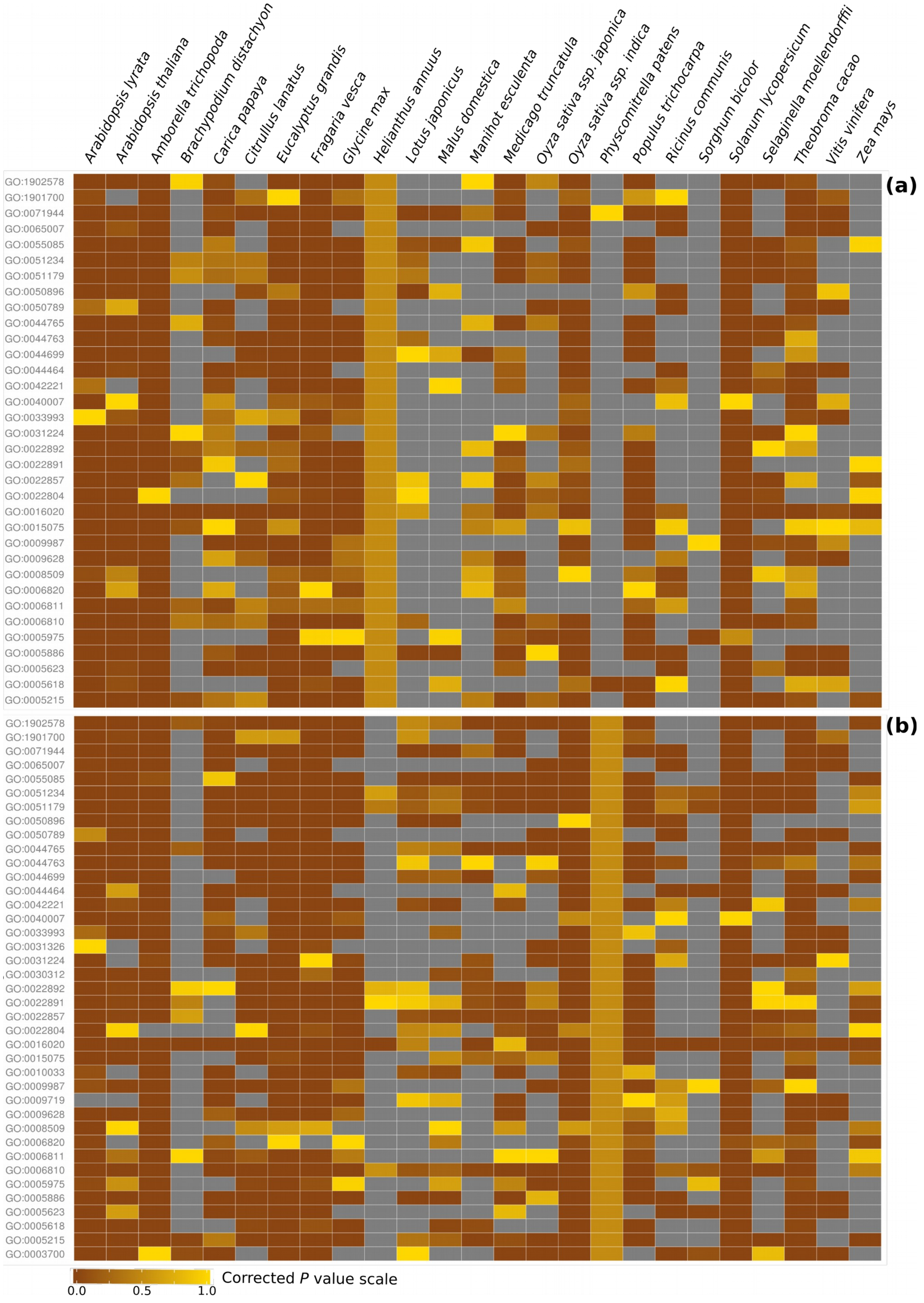

Additional Figure 2

Additional Table 1

Specie	D	Pvalue
Arabidopsis lyrata	0.0228	0.0010
Arabidopsis thaliana	0.0228	0.0012
Amborella trichopoda	0.0228	0.0266
Brachypodium distachyon	0.0228	0.0172
Carica papaya	0.0228	0.1102
Citrullus lanatus	0.0228	0.0399
Eucalyptus grandis	0.0228	0.0000
Fragaria vesca	0.0228	0.0127
Glycine max	0.0741	0.0000
Helianthus annuus	0.0227	0.0000
Lotus japonicus	0.0229	0.0483
Malus domestica	0.1393	0.0000
Manihot esculenta	0.0228	0.0005
Medicago truncatula	0.0228	0.0038
Oryza sativa ssp. indica	0.0228	0.0001
Oryza sativa ssp. japonica	0.0228	0.0019
Physcomitrella patens	0.0228	0.0159
Populus trichocarpa	0.0731	0.0000
Ricinus communis	0.0228	0.0535
Sorghum bicolor	0.0228	0.0081
Solanum lycopersicum	0.0228	0.0004
Selaginella moellendorffii	0.0642	0.0000
Theobroma cacao	0.0228	0.0197
Vitis vinifera	0.0228	0.0064
Zea mays	0.0229	0.0009

Additional Table 2

Specie	TF Duplicates	TF Duplicates by *Ks* ranges											
		0 < Ks ≤ 0.5			0.5 < Ks ≤ 1			1 < Ks ≤ 1.5			1.5 < Ks ≤ 2		
		**WGD**	**Tandem**	**Undefined**	**WGD**	**Tandem**	**Undefined**	**WGD**	**Tandem**	**Undefined**	**WGD**	**Tandem**	**Undefined**
Arabidopsis lyrata	519	23	79	35	116	20	32	73	7	31	60	10	33
Arabidopsis thaliana	532	4	39	49	156	14	28	115	0	14	45	2	66
Amborella trichopoda	61	0	12	12	0	0	3	0	4	3	0	1	26
Brachypodium distachyon	127	3	8	5	9	12	8	9	14	15	13	17	14
Carica papaya	172	0	24	61	0	0	3	1	1	14	1	3	64
Citrullus lanatus	135	0	4	5	1	3	1	5	2	24	16	5	69
Eucalyptus grandis	261	0	95	35	2	17	9	4	8	26	3	8	54
Fragaria vesca	152	5	11	16	5	5	7	11	4	11	25	14	38
Glycine max	1224	579	52	291	95	28	23	49	15	13	53	19	7
Helianthus annuus	305	1	17	61	0	5	72	0	1	48	0	0	31
Lotus japonicus	190	3	10	43	7	9	42	1	2	40	4	7	22
Malus domestica	1009	153	35	713	7	7	39	6	4	14	17	4	10
Manihot esculenta	668	211	8	275	38	4	44	4	4	21	15	2	42
Medicago truncatula	258	18	63	35	19	9	25	20	2	13	19	3	32
Oryza sativa ssp. indica	242	11	34	26	16	7	19	21	10	33	27	5	33
Oryza sativa ssp. japonica	187	5	15	5	18	6	16	19	7	33	29	4	30
Physcomitrella patens	102	1	2	10	5	2	42	3	1	26	1	0	9
Populus trichocarpa	692	434	26	129	12	4	9	15	6	10	29	7	11
Ricinus communis	139	0	5	7	0	2	7	4	0	25	13	4	72
Sorghum bicolor	145	9	20	8	7	5	10	14	8	21	15	9	19
Solanum lycopersicum	400	5	65	42	33	25	62	20	14	38	13	11	72
Selaginella moellendorffii	29	3	3	6	1	1	2	0	0	4	1	0	8
Theobroma cacao	209	5	21	4	0	8	5	21	9	35	47	7	47
Vitis vinifera	377	1	50	25	9	22	34	27	5	81	24	9	90
Zea mays	331	9	21	164	4	3	47	0	5	37	1	11	29

Additional Table 3

Specie	Abbreviation	Release	Source
Arabidopsis lyrata	Aly	Plaza 2.5	ftp.psb.ugent.be/pub/plaza/plaza_public_02_5/
Arabidopsis thaliana	Ath	Plaza 2.5	ftp.psb.ugent.be/pub/plaza/plaza_public_02_5/
Amborella trichopoda	Atr	Plaza 3.0	ftp.psb.ugent.be/pub/plaza/plaza_public_dicots_03/
Brachypodium distachyon	Bdi	Plaza 2.5	ftp.psb.ugent.be/pub/plaza/plaza_public_02_5/
Carica papaya	Cpa	Plaza 2.5	ftp.psb.ugent.be/pub/plaza/plaza_public_02_5/
Citrullus lanatus	Cla	Plaza 3.0	ftp.psb.ugent.be/pub/plaza/plaza_public_dicots_03/
Eucalyptus grandis	Egr	Plaza 3.0	ftp.psb.ugent.be/pub/plaza/plaza_public_dicots_03/
Fragaria vesca	Fve	Plaza 2.5	ftp.psb.ugent.be/pub/plaza/plaza_public_02_5/
Glycine max	Gma	Plaza 2.5	ftp.psb.ugent.be/pub/plaza/plaza_public_02_5/
Helianthus annuus	Hel	HA412 v1.1	http://www.sunflowergenome.org/
Lotus japonicus	Lja	Plaza 2.5	ftp.psb.ugent.be/pub/plaza/plaza_public_02_5/
Malus domestica	Mdo	Plaza 2.5	ftp.psb.ugent.be/pub/plaza/plaza_public_02_5/
Manihot esculenta	Mes	Plaza 2.5	ftp.psb.ugent.be/pub/plaza/plaza_public_02_5/
Medicago truncatula	Mtr	Plaza 2.5	ftp.psb.ugent.be/pub/plaza/plaza_public_02_5/
Oryza sativa ssp. indica	Ind	Plaza 3.0	ftp.psb.ugent.be/pub/plaza/plaza_public_monocots_03/
Oryza sativa ssp. japonica	Osa	Plaza 2.5	ftp.psb.ugent.be/pub/plaza/plaza_public_02_5/
Physcomitrella patens	Ppa	Plaza 2.5	ftp.psb.ugent.be/pub/plaza/plaza_public_02_5/
Populus trichocarpa	Ptr	Plaza 2.5	ftp.psb.ugent.be/pub/plaza/plaza_public_02_5/
Ricinus communis	Rco	Plaza 2.5	ftp.psb.ugent.be/pub/plaza/plaza_public_02_5/
Sorghum bicolor	Sbi	Plaza 2.5	ftp.psb.ugent.be/pub/plaza/plaza_public_02_5/
Solanum lycopersicum	Sly	Plaza 3.0	ftp.psb.ugent.be/pub/plaza/plaza_public_dicots_03/
Selaginella moellendorffii	Smo	Plaza 2.5	ftp.psb.ugent.be/pub/plaza/plaza_public_02_5/
Theobroma cacao	Tca	Plaza 2.5	ftp.psb.ugent.be/pub/plaza/plaza_public_02_5/
Vitis vinifera	Vvi	Plaza 2.5	ftp.psb.ugent.be/pub/plaza/plaza_public_02_5/
Zea mays	Zma	Plaza 2.5	ftp.psb.ugent.be/pub/plaza/plaza_public_02_5/
